# Talarodiolide, a New 12-Membered Macrodiolide, and GC/MS Investigation of Culture Filtrate and Mycelial Extracts of *Talaromyces pinophilus*

**DOI:** 10.3390/molecules23040950

**Published:** 2018-04-19

**Authors:** Maria Michela Salvatore, Marina DellaGreca, Rosario Nicoletti, Francesco Salvatore, Francesco Vinale, Daniele Naviglio, Anna Andolfi

**Affiliations:** 1 Department of Chemical Sciences, University of Naples ‘Federico II’, 80126 Naples, Italy; mariamichela.salvatore@unina.it (M.M.S.); dellagre@unina.it (M.D.); frsalvat@unina.it (F.S.); naviglio@unina.it (D.N.); 2 Council for Agricultural Research and Agricultural Economy Analysis, 00184 Rome, Italy; rosario.nicoletti@crea.gov.it; 3 Department of Agriculture, University of Naples ‘Federico II’, 80055 Portici, Italy; 4 Institute for Sustainable Plant Protection, National Research Council, 80055 Portici (NA), Italy; francesco.vinale@ipsp.cnr.it

**Keywords:** *Talaromyces pinophilus*, talarodiolide, macrodiolides, GC/MS, secondary metabolites

## Abstract

Talarodiolide, a new 12-membered macrodiolide, was isolated and characterized from the culture filtrate of strain LT6 of *Talaromyces pinophilus*. The structure of (Z)-4,10-dimethyl-1,7-dioxa-cyclododeca-3,9-diene-2,8-dione was assigned essentially based on NMR and MS data. Furthermore, several known compounds were isolated and identified in the crude extract of the culture filtrate and mycelium of this strain. EI mass spectrum at 70 eV of all isolated metabolites was acquired and compiled in a custom GC/MS library to be employed to detect metabolites in the crude extracts.

## 1. Introduction

With a widespread occurrence in very diverse environmental contexts, from the soil to the sea [1,2,3], the species *Talaromyces pinophilus* (=*Penicillium pinophilum*) (Eurotiales: Trichocomaceae) has received increasing attention in mycological research for its ability to act as a fungal antagonist and plant-growth promoter [1,4,5], and for possible biotechnological applications based on the production of enzymes [6,7] and bioactive metabolites [8,9,10].

Two strains (LT4 and LT6), possibly deriving from the same wild clone since they were both recovered from the rhizosphere of a tobacco plant cropped near Lecce (Apulia, Southern Italy), have been particularly studied in our laboratories after they were shown to produce a novel fungitoxic and cytostatic compound named 3-*O*-methylfunicone (OMF) [1,11]. OMF is part of a homogeneous family comprising about 20 structurally related secondary metabolites which have been mainly characterized from cultures of *Talaromyces* strains [12]. It has notable antitumor properties based on several biomolecular mechanisms of action resulting from a series of preclinical assays [13,14,15,16,17]. Although it represents the main extrolite produced by our strains, other funicone variants have been occasionally extracted [18,19], indicating that some factors act during the culturing cycle which may lead to the accumulation of intermediate or side products. Within our recent activity aiming at the standardization of OMF production, additional compounds were detected from cultures of strain LT6. Among them, a new product with an unusual structure for a natural compound, namely talarodiolide, was purified from its culture filtrates. Furthermore, the present paper reports findings from the first GC/MS-based investigation on secondary metabolites in culture filtrate and mycelial extracts of *T. pinophilus*.

## 2. Results

### 2.1. Isolation and Identification of Metabolites

**Figure 1:**
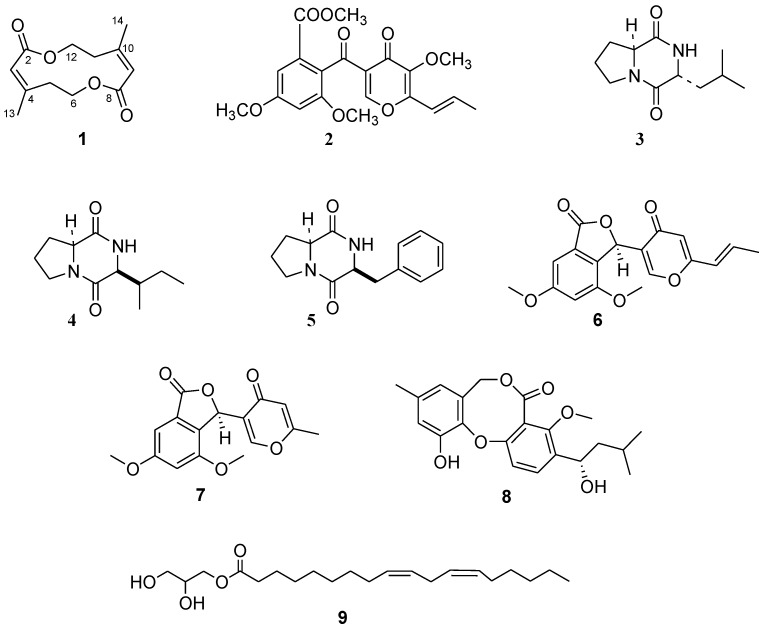
Structures of talarodiolide, 3-*O*-methylfunicone, *cyclo*-(*S*-Pro-*R*-Leu), *cyclo*-(*S*-Pro-*S*-Ile), *cyclo*-(*S*-Pro-*S*-Phe), vermistatin, penisimplicissin, penicillide, and 1-glycerol-linoleate (**1**–**9**), compounds produced by *Talaromyces pinophilus* LT6, isolated by preparative chromatographic methods and identified by spectroscopic and MS techniques.

The crude CHCl_3_ extract from the culture filtrates of *T. pinophilus* strain LT6 was purified by combined column (CC) and thin layer chromatography (TLC), leading to isolation of one new (**1**, Figure 1) and four known compounds (**2**–**5**, Figure 1). Structures of known compounds were confirmed by comparison of data obtained from OR, ^1^H and ^13^C-NMR , and ESI-TOF MS with those reported in the literature for OMF [11], *cyclo*-(*S*-Pro-*R*-Leu), *cyclo*-(*S*-Pro-*S*-Ile) [20], and *cyclo*-(*S*-Pro-*S*-Phe) [21] (**2**–**5**).

Compound **1**, isolated as amorphous solid, has a molecular weight of 224 *m*/*z* accounting for a molecular formula of C_12_H_16_O_4_ and the index of hydrogen deficiency is five as deduced from ESI-TOF MS. The ^1^H-NMR spectrum (Table 1 and Figure S1) revealed one broad singlet methyl, one broad triplet and one triplet in aliphatic region, and a broad singlet of olefinic signals. In the ^13^C-NMR spectrum (Table 1 and Figure S5), only six carbon signals were present indicating a highly symmetric molecule. The ^1^H and ^13^C resonances of **1** were assigned by combination of COSY and HSQC experiments. The COSY experiment showed homocorrelations among the olefinic proton at δ 5.84 with the methyl at δ 2.03 and methylene at δ 2.40, the latter of which was also correlated with methylene at δ 4.40. The HSQC (Figure S3) spectrum showed correlations of methyl at δ 2.03 with carbon at δ 22.4, two methylenes at δ 2.40 and 4.40 with carbons at δ 29.2 and 65.8, respectively, and one methine at δ 5.84 with carbon 116.8. The carbons at δ 164.6 and 157.7 were assigned to a carboxyl group and substituted sp^2^ carbon, respectively. According to the structure in the HMBC (Figure S4) spectrum, the H_2_-6/H_2_-12 protons were correlated to the C-8/C-2 at 164.4, C-4/C-10 at 157.7 and C-5/C-11 at 29.2. Furthermore, the H_3_-13/H_3_-14 protons were correlated to C-3/C-9, C-4/C-10 and C-5/C-11 carbons. The analysis of NOESY (Figure S6) spectrum evidenced NOE of the methyl at δ 2.03 and olefinic H-3 proton indicating a Z configuration at double bond.

These results and the molecular formula of C_12_H_16_O_4_ suggest that **1** is a symmetrical macrodiolides, (Z)-4,10-dimethyl-1,7-dioxa-cyclododeca-3,9-diene-2,8-dione. This structure was confirmed by data from ESI-TOF MS recorded in positive mode. The spectrum showed the sodiated dimeric, dimeric, sodiated and pseudomolecular ions [2M + Na]^+^, [2M + H]^+^, [M + Na]^+^, and [M + H]^+^ at *m/z* 471, 449, 247, and 225, respectively. 

Symmetric macrodiolides have been reported from many natural sources, and displayed some interesting effects, such as antibacterial, antifungal and cytotoxic activities ([22] and literature therein). However, in the light of the current knowledge, no 12-membered macrodiolide has been isolated from natural sources so far. 

In addition, the production of secondary metabolites by *T. pinophilus* LT6 was investigated after extraction of mycelium. Extraction and purification procedures (CC and TLC) afforded the isolation of OMF (**2**), and other known compounds identified as vermistatin (**6**) [23], penisimplicissin (**7**) [24], penicillide (**8**) [25], and 1-glycerol-linoleate (**9**) (Figure 1). In the case of **9**, preliminary NMR investigation showed typical signals of monoglycerides of polyunsaturated fatty acids [26]. GC/MS measurements confirmed NMR data and unequivocally revealed the presence of this monoglyceride by comparing its mass spectrum with the reference mass spectra gathered in NIST 14 Mass Spectral library (2014) [27].

### 2.2. GC/MS Analysis 

In this study, an EI mass spectrum at 70 eV of all isolated metabolites was acquired and compiled in a custom MS target library to be employed to detect metabolites separated in the crude extracts. GC/MS measurements served several purposes within our strategy. First, when the mass spectrum of the metabolite could be retrieved from a MS database, the acquired mass spectrum provided a definitive proof of its identity, as in the case of *cyclo*-(*S*-Pro-*R*-Leu). 

When no mass spectrum satisfactorily matches the acquired mass spectrum could be inferred from a database, the unknown metabolite had to be otherwise identified (e.g., via ESI-TOF MS and ^1^H/^13^C-NMR mono- and bi-dimensional), but interpretation of the acquired mass spectrum served as a guide in the identification process by setting restrictions on possible structures. 

In all cases, the acquired mass spectrum was incorporated into the custom MS library to be used for interpreting GC/MS measurements to be performed directly on samples of mycelium and culture filtrates extracts obtained. Table 2 shows data collected via GC/MS of the identified metabolites.

**Figure 2:**
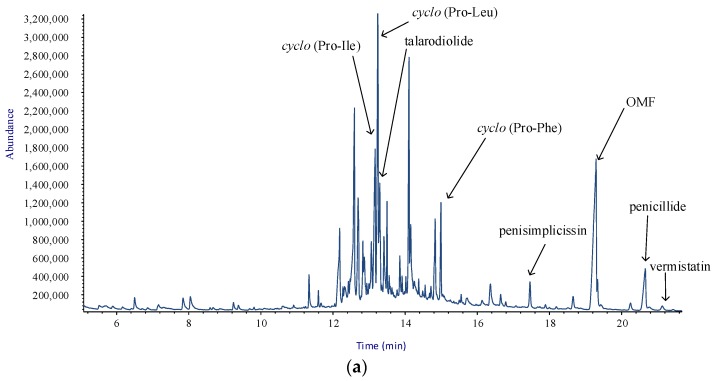

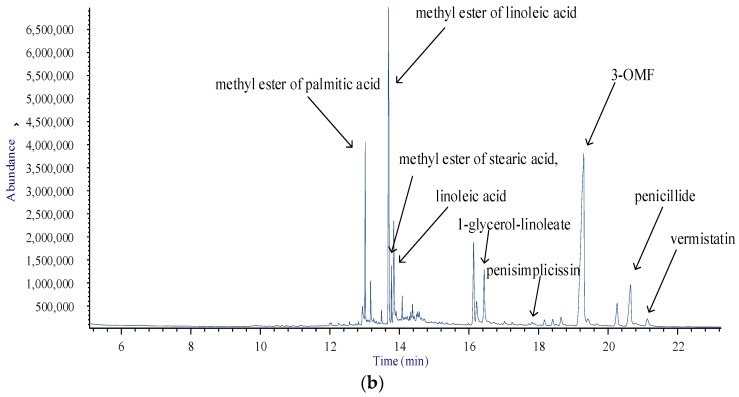
Annotated total ion chromatograms (TICs) acquired by: culture filtrate extract (**a**); and mycelial extract (**b**) of *T. pinophylus*.

Figure 2a,b shows the total ion chromatograms (TICs) of the extracts of culture filtrate and mycelium, respectively. 

Apart from the isolated metabolites, Figure 2b shows the presence of some fatty acids and their methyl esters in the mycelial extract. In fact, due to the high sensitivity of this technique, GC/MS was able to detect them, combining the retention indices and the reference mass spectra gathered in NIST 14 Mass Spectral library (2014) [27]. 

Within the framework of the overall strategy, a very important outcome of the procedures arises from the fact that crude extracts were analyzed by GC/MS to check the presence of the isolated metabolites. Notwithstanding some metabolites were not isolated from the culture filtrate, AMDIS attributes peaks in the TIC, as in the case of penicillide, vermistatin and penisimplicissin. Hence, GC/MS analysis is very useful in assessing the possible diversity in the pattern of metabolites extracted from the different sources. With exception of talarodiolide, 1-glycerol-linoleate and the diketopiperazines, all metabolites were detected in both crude extracts, while fatty acids and their esters (**10**–**13**) are present in the mycelial extract only. This is in line with the reported occurrence of the latter compounds in the cell membrane of fungi [28]. 

## 3. Materials and Methods 

### 3.1. General Experimental Procedures

Optical rotations were measured in CHCl_3_, CH_3_OH, and C_2_H_5_OH on a Jasco P-1010 digital polarimeter; ^1^H and ^13^C-NMR spectra were recorded at 400/100 MHz in CDCl_3_ or in CD_3_OD on Bruker (Bremen, Germany) spectrometers. The same solvent was used as internal standard. 2D NMR experiments were performed using Bruker microprograms. ESI-TOF mass spectra have been measured on an Agilent Technologies QTOF 6230 in the positive ion mode (Milan, Italy). 

Analytical and preparative TLC were performed on silica gel plates (Kieselgel 60, F254, 0.25 and 0.5 mm, respectively) (Merck, Darmstadt, Germany). The spots were visualized by exposure to UV radiation (253), or by spraying first with 10% H_2_SO_4_ in MeOH followed by heating at 110 °C for 10 min. Column chromatography was performed on silica gel column (Merck, Kieselgel 60, 0.063–0.200 mm).

GC/MS measurements were performed with an Agilent 6850 GC equipped with an HP-5MS capillary column (5% phenyl methyl polysiloxane stationary phase) and the Agilent 5973 Inert MS detector (used in the scan mode). Helium was employed as the carrier gas, at a flow rate of 1 mL/min. The injector temperature was 250 °C and during the run a temperature ramp raised the column temperature from 70 °C to 280 °C: 70 °C for 1 min; 10 °C min^−1^ until reaching 170 °C; and 30 °C min^−1^ until reaching 280 °C. Then it was held at 280 °C for 5 min. The electron impact (EI) ion source was operated at 70 eV and at 200 °C. The quadrupole mass filter was kept at 250 °C and was programmed to scan the range 45–550 *m/z* at a frequency of 3.9 Hz.

### 3.2. Culture Filtrate Preparation

Liquid cultures were prepared by inoculating mycelial plugs from actively growing cultures of strain LT6 in 1 L-Erlenmayer flasks containing 500 mL potato–dextrose broth (PDB, Himedia) which were kept in darkness on stationary phase at 25 °C. After 21 days, cultures were filtered at 0.45 µm, and the culture filtrates were concentrated in a lyophilizer until reduction to 1/10 of the starting volume. The mycelial cake floating on the broth was collected separately and stored at −20 °C.

### 3.3. Extraction and Isolation of Metabolites from Liquid Cultures

The freeze-dried culture filtrates (6 L) were dissolved in 600 mL of pure water (pH 4) and extracted with same volume of CHCl_3_ for three times. The organic extracts were combined, dried on Na_2_SO_4_, and evaporated under reduced pressure to give a yellowish oil residue (75.3 mg).

The residue was submitted to fractionation on silica gel column (1.5 × 30 cm i. d.), eluted with CHCl_3_/*iso*-PrOH (98:2, *v/v*). Seven homogeneous fraction groups were collected (A 0.7 mg, B 2.7 mg, C 9.5 mg, D 0.8 mg, E 3.4 mg, F 9.3 mg, G 8.2 mg).The residue of fraction C was purified by TLC on silica gel eluted with *n*-hexane-acetone (6:4, *v/v*) yielding an amorphous solid, talarodiolide (**1**, 1.5 mg, R_f_ 0.41 on TLC on silica gel eluent *n*-hexane-acetone (6:4, *v/v*)), and a crystalline solid, OMF (**2**, 3.5 mg, R_f_ 0.47 on TLC on silica gel eluent *n*-hexane-acetone (6:4, *v/v*)).The residue of the fraction F was further purified by TLC on silica gel eluted with CHCl_3_/*iso*-PrOH (95:5, *v/v*) giving as amorphous solids: *cyclo*-(*S*-Pro-*R*-Leu) (**3**, 1.0 mg, R_f_ 0.49 on TLC on silica gel eluent CHCl_3_-*i*-PrOH (95:5, *v/v*)), *cyclo-*(*S*-Pro-*S*-Ile) (**4**, 2.3 mg, R_f_ 0.35 on TLC on silica gel eluent CHCl_3_-*i*-PrOH (95:5, *v/v*)), and *cyclo-*(*S*-Pro-*S*-Phe) (**5**, 1.5 mg, R_f_ 0.32 on TLC on silica gel eluent CHCl_3_-*i*-PrOH (95:5, *v/v*)). 

### 3.4. Extraction and Isolation of Metabolites from Mycelium

Fresh mycelium was homogenized in a mixer with 440 mL of MeOH-H_2_O (NaCl 1%) mixture (55:45, *v/v*). The suspension was stirred in the dark at room temperature for 4 h. After this period, the suspension was centrifuged (40 min at 7000 rpm, 10 °C) and separated from the supernatant. The residue was overnight extracted with 250 mL of the mixture reported above. The suspension was centrifuged, and both supernatants were combined for the subsequent extraction with CHCl_3_. The organic extracts were combined, dried on anhydrous Na_2_SO_4_, and evaporated under reduced pressure yielding crude extract as a red oil (230.2 mg). The extract was fractionated by CC on silica gel (1.5 × 40 cm i. d.), eluting with CHCl_3_/*iso*-PrOH (97:3, *v/v*). The last fraction was eluted with MeOH. Seven homogeneous fraction groups were collected (A 16.0 mg, B 16.4 mg, C 12.2 mg, D 14.2 mg, E 9.8 mg, F 29.1 mg, G 66.2 mg). The residue of fraction B was identified as OMF (**2**). Fraction C was purified by TLC on silica gel eluted with *n*-hexane/acetone (6:4, *v/v*) to afford a further amount of OMF (5.6 mg), a crystalline compound identified as vermistatin (**6**, 1.5 mg, R_f_ 0.37 on TLC on silica gel eluent *n*-hexane-acetone (6:4, *v/v*)), and an amorphous solid identified as penisimplicissin (**7**, 0.5, mg, R_f_ 0.29 on TLC on silica gel eluting with *n*-hexane-acetone (6:4, *v/v*)). Fraction D was purified using the same condition described for C giving penicillide (**8**, 6.9, mg, R_f_ 0.29 on TLC on silica gel eluent *n*-hexane-acetone (6:4, *v/v*)) as amorphous solid. Finally, the residue of fraction F was further purified on TLC on silica gel eluting with CHCl_3_/*iso*-PrOH (9:1, *v/v*) giving 1-glycerol-linoleate (**9**, 1.5 mg, R_f_ 0.40 on TLC on silica gel eluent CHCl_3_/*iso*-PrOH (9:1, *v/v*)) as soft solid.

*Talarodiolide* (**1**): amorphous solid; UV (CH_3_CN) λ_max_ (log ε) 260 (3.15); HRESIMS (+): 471.1990 ([calcd. 471.1995 for C_24_H_32_O_8_Na 2M + Na]^+^), 449.2182 ([calcd. 449.2175 for C_24_H_33_O_8_ 2M + H]^+^), 247.0950 ([calcd. 247.0941 for C_12_H_16_O_4_Na M + Na]^+^), 225.1118 ([calcd. 225.1127 for C_12_H_17_O_4_ M + H]^+^); ^1^H-NMR (CDCl_3_, 400 MHz) and ^13^C-NMR (CDCl_3_, 100 MHz) data: see Table 1.

*Cyclo-(S-Pro-R-Leu)* (**3**): amorphous solid; [α]_D_ −88° (c = 0.12, C_2_H_5_OH); HRESIMS (+): 443.2636 ([calcd. 443.2629 for C_22_H_36_N_4_O_4_Na 2M + Na]^+^), 233.1269 ([calcd. 233.1260 for C_11_H_18_N_2_O_2_Na M + Na]^+^), 211.1448 ([calcd. 211.1441 for C_11_H_19_N_2_O_2_ M + H]^+^). Optical rotation and NMR data are in agreement with those previously reported [20].

*Cyclo-(S-Pro-S-Ile*) (**4**): amorphous solid; [α]_D_ −193° (c = 0.11, C_2_H_5_OH); HRESIMS (+): 233.1272 ([calcd. 233.1260 for C_11_H_18_N_2_O_2_Na M + Na]^+^), 211.1451 ([calcd. 211.1441 for C_11_H_19_N_2_O_2_ M + H]^+^); Optical rotation and NMR data are in agreement with those previously reported [20].

*Cyclo-(S-Pro-S-Phe)* (**5**): amorphous solid; [α]_D_ −65° (c = 0.10, CH_3_OH); HRESIMS (+): 267.1115 ([calcd. 267.1109 for C_14_H_16_N_2_O_2_Na M + Na]^+^), 245.1296 ([calcd. 245.1290 for C_14_H_17_N_2_O_2_ M + H]^+^); Optical rotation and NMR data are in agreement with those previously reported [21].

*Vermistatin* (**6**): crystalline compound; [α]_D_ −6° (c = 0.14, CHCl_3_); HRESIMS (+): 351.0841 ([calcd. 351.0845 for C_18_H_16_O_6_Na M + Na]^+^), 329.1025 ([calcd. 329.1029 for C_18_H_17_O_6_ M + H]^+^). Optical rotation and NMR data are in agreement with those previously reported [23].

*Penisimplicissin* (**7**): amorphous solid; [α]_D_ −112° (c = 0.15, CHCl_3_); HRESIMS (+): 627.1475 ([calcd. 627.1473 for C_32_H_28_O_12_Na 2M + Na]^+^), 325.0686 ([calcd. 325.0683 for C_16_H_14_O_6_Na M + Na]^+^), 303.0869 ([calcd. 303.0863 for C_16_H_15_O_6_ M + H]^+^). Optical rotation and NMR data are in agreement with those previously reported [24].

*Penicillide* (**8**): amorphous solid; [α]_D_ +6° (c = 0.16, CHCl_3_); HRESIMS (+): 409.2565 ([calcd. 409.1627 for C_22_H_26_O_6_Na M + Na]^+^), 371.1493 ([calcd. 371.1489 for C_21_H_23_O_6_ M − CH_3_]^+^), 359 [M + H − CO]^+^. Optical rotation and NMR data are in agreement with those previously reported ([25] and literature therein).

### 3.5. GC/MS Analysis

GC/MS data were acquired on crude extracts or isolated metabolites. The metabolite identities were confirmed acquiring mass spectra of pure compounds and high-quality mass spectra were obtained employing the National Institute of Standards and Technology (NIST) deconvolution software Automatic Mass spectral Deconvolution & Identification System (AMDIS) [29,30]. Mass spectra were stored in the custom MS target library of metabolites [31]. Fatty acids and esters of fatty acids were identified by comparing their mass spectra with spectra of pure compounds gathered in the database NIST 14 Mass Spectral library [27] by employing the NIST Mass Spectral Search Program v.2.0g [32].

## 4. Conclusions

The present paper describes the isolation and structural characterization of the first 12-membered macrodiolide, named talarodiolide, from the culture filtrate of strain LT6 of *T. pinophilus*. We expect we will be able to isolate sufficient amount of talarodiolide for biological studies. Furthermore, the identification of metabolites present in culture filtrate and mycelial extracts of this strain was carried out with the support of a custom GC/MS library mainly built after isolation and identification of metabolites via NMR spectroscopy. This strategy represents a suitable approach for the screening, with high confidence, of several metabolites present in crude extracts and future works will focus on testing the effects of experimental conditions (i.e., media composition, co-cultivation with other microbes, etc.) on the production of secondary metabolites by strains of *T. pinophilus*.

## Figures and Tables

**Table 1:** NMR data and HMBC correlations for talarodiolide (**1**) recorded in CDCl_3_.

Position	δ_C_	δ_H_ (*J* in Hz)	HMBC
2, 8	164.6 C	-	
3, 9	116.8 CH	5.84, brs	
4,10	157.7 C	-	
5, 11	29.2 CH_2_	2.40, brt, 6.3	
6, 12	65.8 CH_2_	4.40, t, 6.3	C-8/C-2,C-4/C-10, C-5/C-11
13, 14	22.4 CH_3_	2.03, brs	C-3/C-9, C-4/C-10, C-5/C-11

**Table 2:** GC/MS analysis of the crude extract of culture filtrate (A) and mycelium (B) of *T. pinophilus* LT6.

Metabolite	Code	Diagnostic Ions *m*/*z* (Abundance)	RI	A% of Total Ion Current	B% of Total Ion Current
Talarodiolide	**1**	224 [M]•^+^ (5), 209 [M − Me]^+^ (4), 194 [M − 2Me]^+^ (35), 149 [M − 2Me − CO_2_ − O]^+^ (60), 70 [M − C_8_H_9_O_3_]^+^ (100)	2064	3.55	
3-*O*-Methylfunicone	**2**	388 [M]•^+^ (40), 373 [M − Me]^+^ (15), 357 [M − 2Me]^+^, 223 [M − C_9_O_3_H_9_]^+^ (65), 192 [M − 2Me − C_9_O_3_H_9_]^+^ (100)	3006	15.26	38.12
*Cyclo-*(Pro-Leu)	**3**	195 [M − Me]^+^ (5), 154 [M − C_4_H_9_]^+^ (100), 125 [M − C_6_H_13_]^+^ (15), 111 [M − C_7_H_15_]^+^ (3), 70 [M − C_7_NO_2_H_11_]^+^ (75)	2068	11.06	
*Cyclo-*(Pro-Ile)	**4**	154 [M − C_4_H_9_]^+^ (100), 125 [M − C_6_H_13_]^+^ (120), 111 [M − C_7_H_15_]^+^ (5), 70 [M − C_7_NO_2_H_11_]^+^ (65)	2039	6.90	
*Cyclo*-(Pro-Phe)	**5**	244 [M]•^+^ (34), 215 [M − C_2_H_4_]^+^ (3), 153 [M − C_6_H_5_ − CH_2_] (28), 125 [M − C_3_H_6_ − C_6_H_5_] (100)	2443	2.93	
Vermistatin	**6**	328 [M]•^+^ (100), 313 [M − Me]^+^ (10), 285 [M − Me − C_2_H_4_]^+^ (48), 165 [M − C_2_H_4_ − C_8_O_2_H_8_]^+^ (43)	3105	0.424	1.124
Penisimplicissin	**7**	302 [M]•^+^ (100), 287 [M − Me]^+^, 273 [M − 2Me]^+^ (17), 175 [M − Me − C_6_H_7_O_2_] (14), 165 [M − C_8_O_2_H_8_]^+^ (47)	2835	1.328	0.39
Penicillide	**8**	372 [M − Me]^+^ (16), 269 [M − 2Me − C_5_OH_10_] (100), 253 [M − Me − OCH_3_ − C_5_OH_10_] (20)	3103	3.64	6.71
1-glycerol-linoleate	**9**	354 [M]•^+^ (4), 336 [M − OH]^+^, 262 [M − C_3_O_3_H_7_]^+^ (63), 234 [M − C_4_O_4_H_7_]^+^ (12)	2076		4.19
Methyl ester of palmitic acid	**10**	[27]	2020		5.73
Methyl ester of linoleic acid	**11**	[27]	2146		17.211
Methyl ester of stearic acid	**12**	[27]	2158		1.76
Linoleic acid	**13**	[27]	2169		6.64

## Supplementary Materials

The following are available online. NMR spectra of talarodiolide; EI mass spectra at 70 eV of metabolites from *T. pinophilus*.
