# Supplementary material for: Talarodiolide, a New 12-Membered Macrodiolide, and GC/MS Investigation of Culture Filtrate and Mycelial Extracts of Talaromyces pinophilus
Source: Molecules. 2018 Apr 19;23(4):950. doi: 10.3390/molecules23040950 (PMC6017668; doi:10.3390/molecules23040950)

Supplementary Materials

Talarodiolide, the new 12-membered macrodiolide, and GC/MS investigation of mycelial and culture filtrate extracts of *Talaromyces pinophilus*

Maria Michela Salvatore [1], Marina DellaGreca [1], Rosario Nicoletti [2,3], Francesco Salvatore[1], Daniele Naviglio[1] and Anna Andolfi [1,*]

[1] Department of Chemical Sciences, University of Naples 'Federico II', Naples 80126, Italy; mariamichela.salvatore@unina.it (M.M.S.); dellagre@unina.it (M.DG.); frsalvat@unina.it (F.S.); naviglio@unina.it (D.N.)

[2] Council for Agricultural Research and Agricultural Economy Analysis, Rome 00184, Italy

[3] Department of Agriculture, University of Naples 'Federico II', Portici 80055, Italy; rosario.nicoletti@crea.gov.it

[*] Correspondence: andolfi@unina.it (A.A); Tel.: +39-081-2539179

Figure S1. ^{1}H NMR spectrum of talarodiolide (**1**) recorded in $CDCl_3$ at 400 MHz

Figure S2. COSY spectrum of talarodiolide (**1**) recorded in $CDCl_3$ at 400 MHz

Figure S3. HSQC spectrum of talarodiolide (**1**) recorded in $CDCl_3$ at 400 MHz

Figure S4. HMBC spectrum of talarodiolide (**1**) recorded in $CDCl_3$ at 400 MHz

Figure S5. ^{13}C NMR spectrum of talarodiolide (**1**) recorded in $CDCl_3$ at 100 MHz

Figure S6. NOESY spectrum of talarodiolide (**1**) recorded in $CDCl_3$ at 400 MHz

Table S1. EI mass spectra at 70eV of identified metabolites.

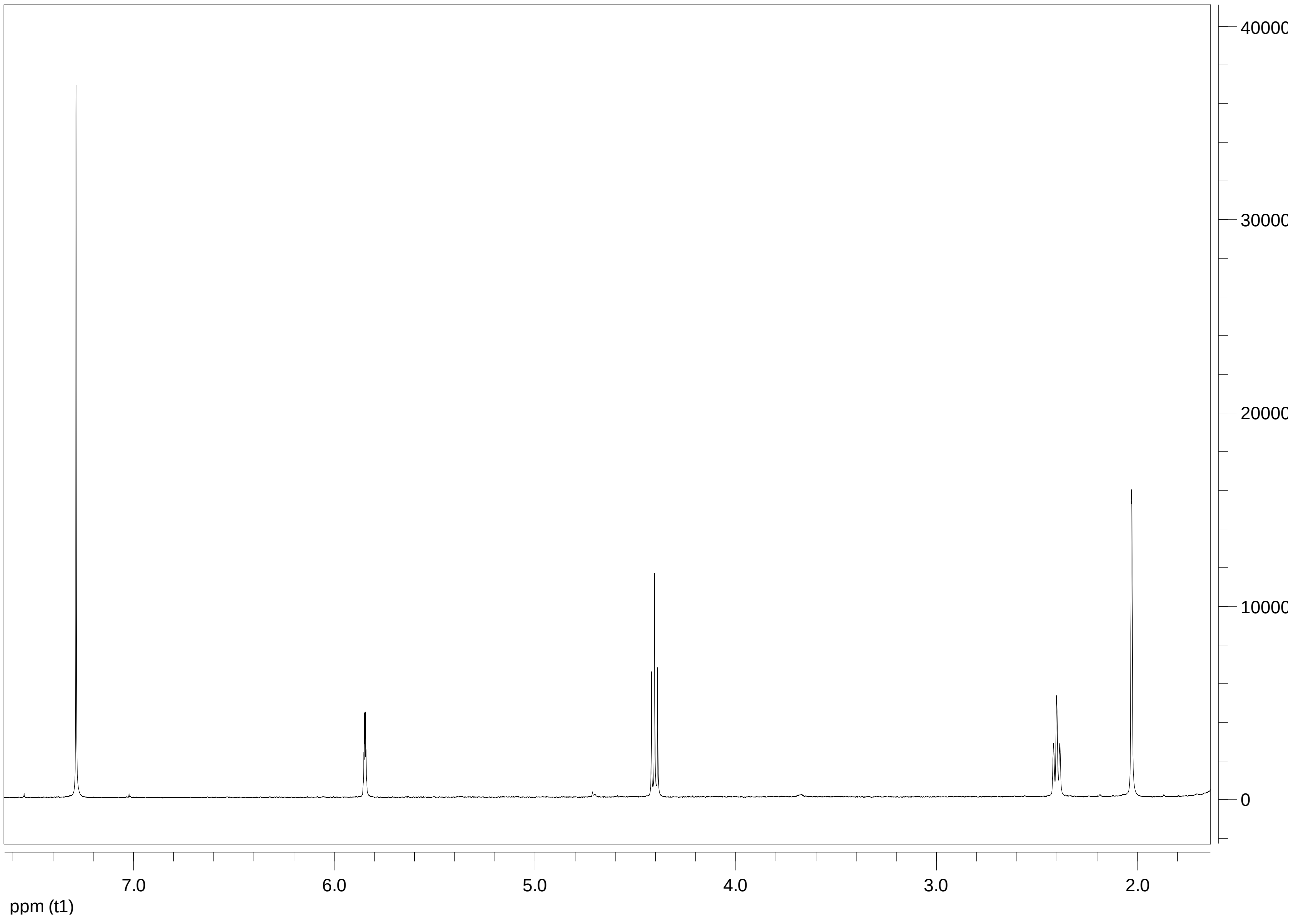

Figure S1. ^{1}H NMR spectrum of talarodiolide (**1**) recorded in $CDCl_3$ at 400 MHz

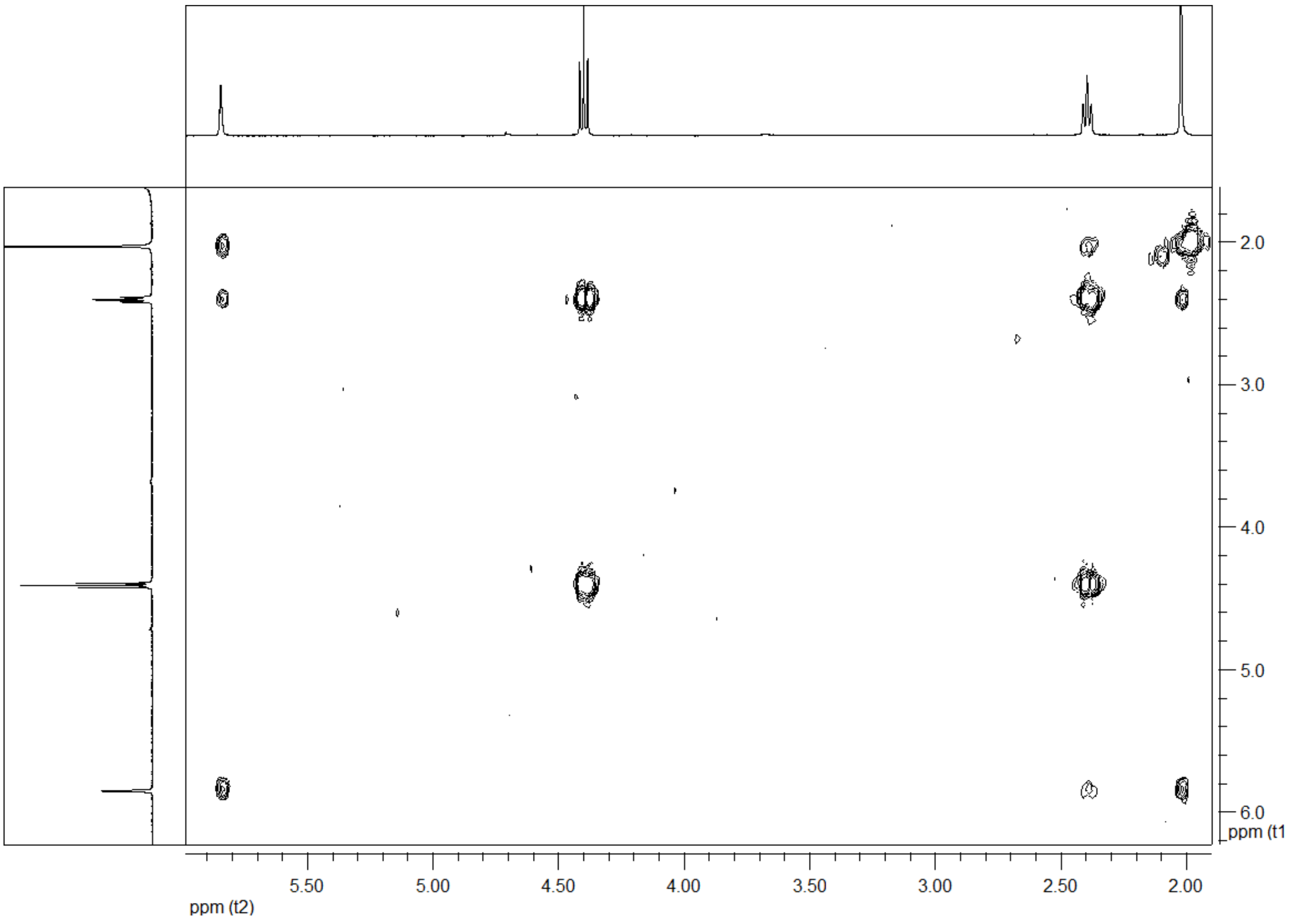

Figure S2. COSY spectrum of talarodiolide (**1**) recorded in $CDCl_3$ at 400 MHz

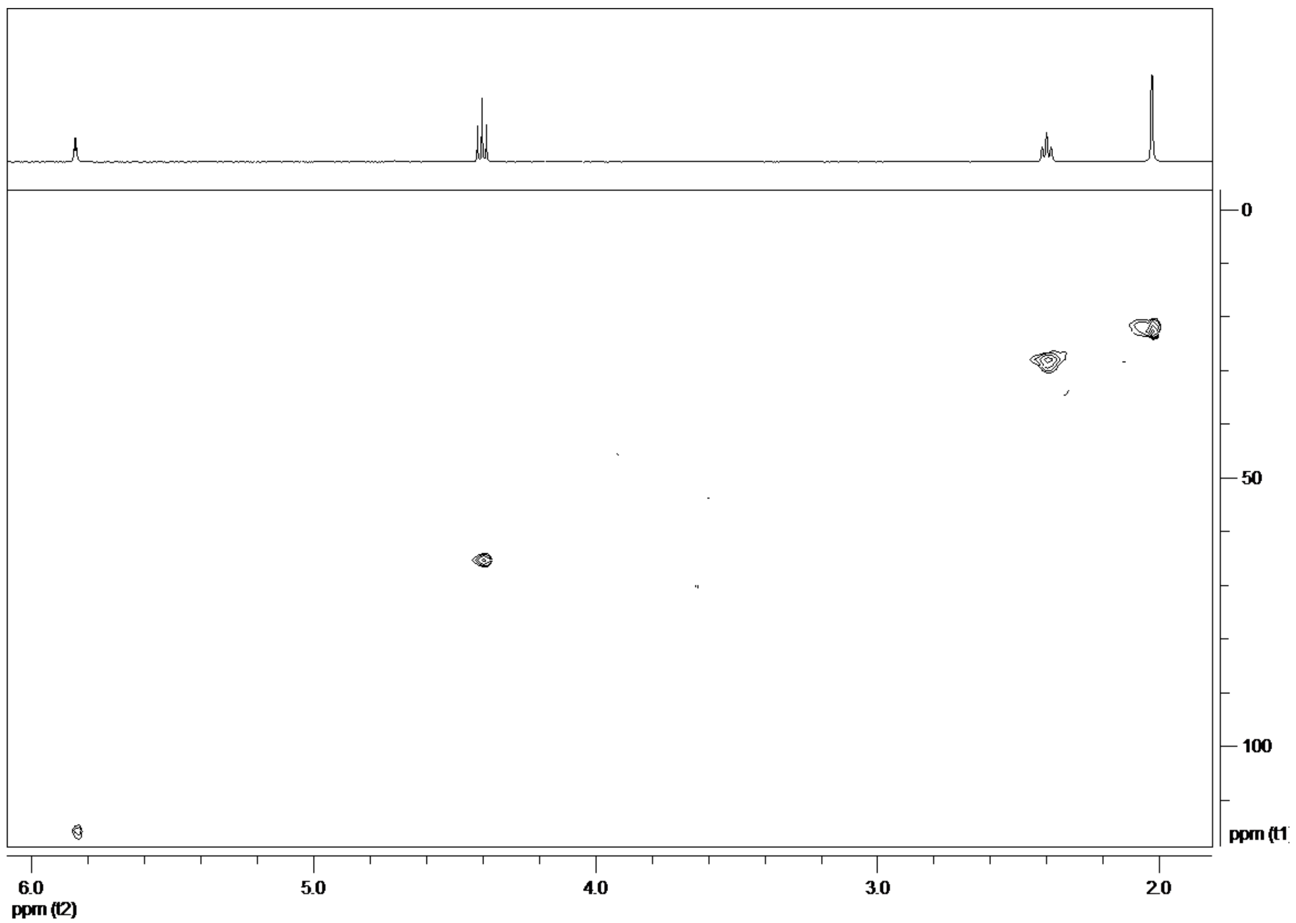

Figure S3. HSQC spectrum of talarodiolide (**1**) recorded in $CDCl_3$ at 400 MHz

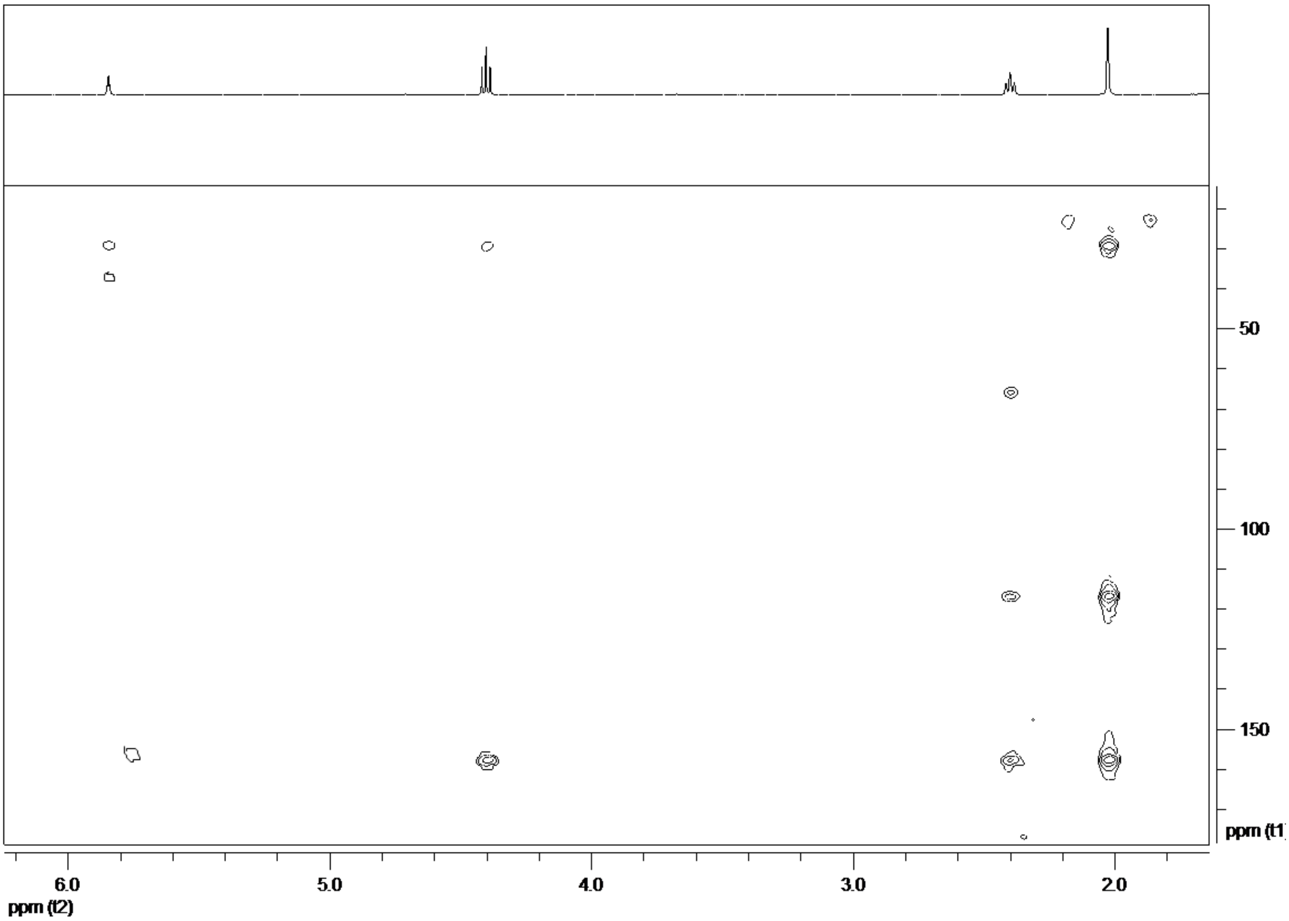

Figure S4. HMBC spectrum of talarodiolide (**1**) recorded in $CDCl_3$ at 400 MHz

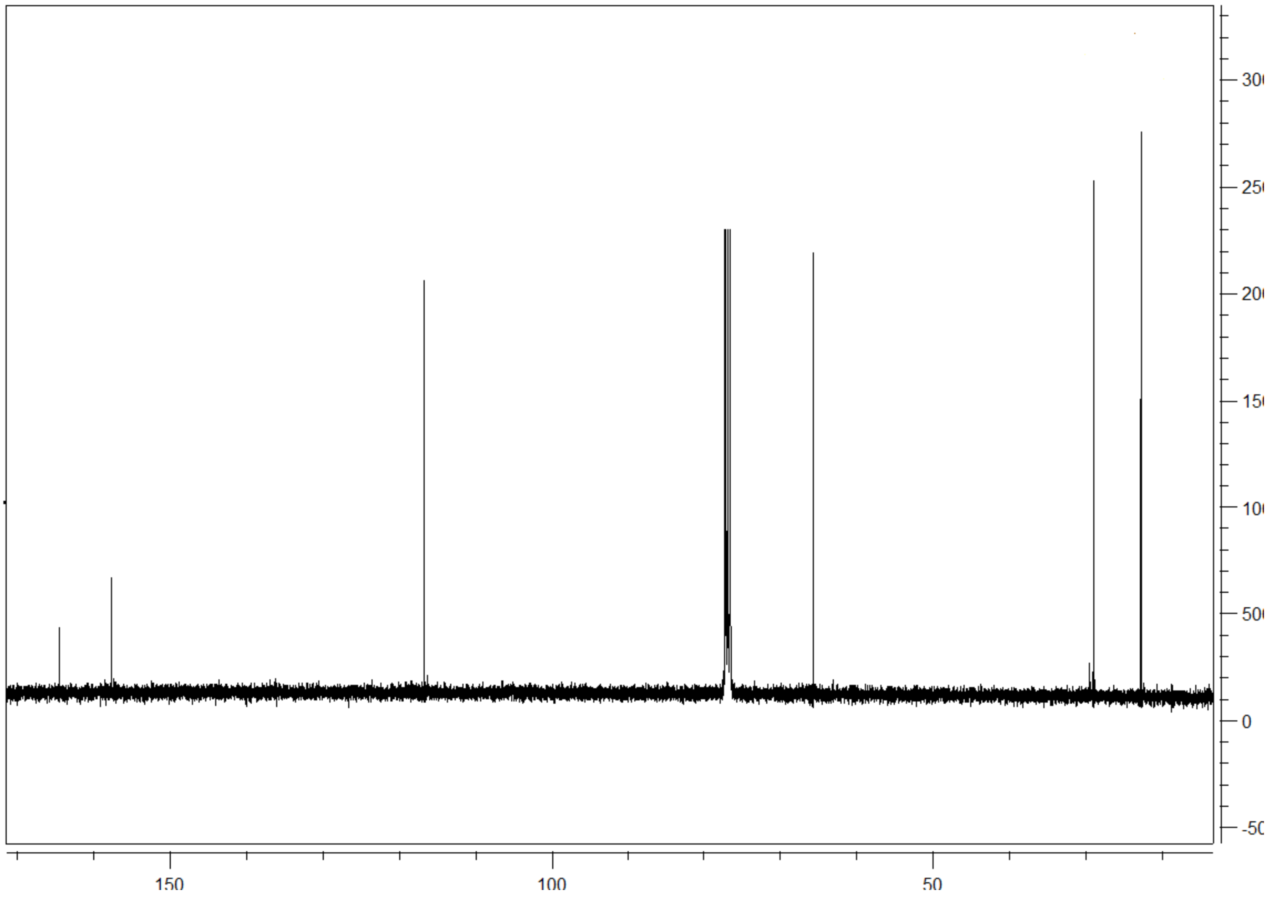

Figure S5. ^{13}C NMR spectrum of talarodiolide (**1**) recorded in $CDCl_3$ at 100 MHz

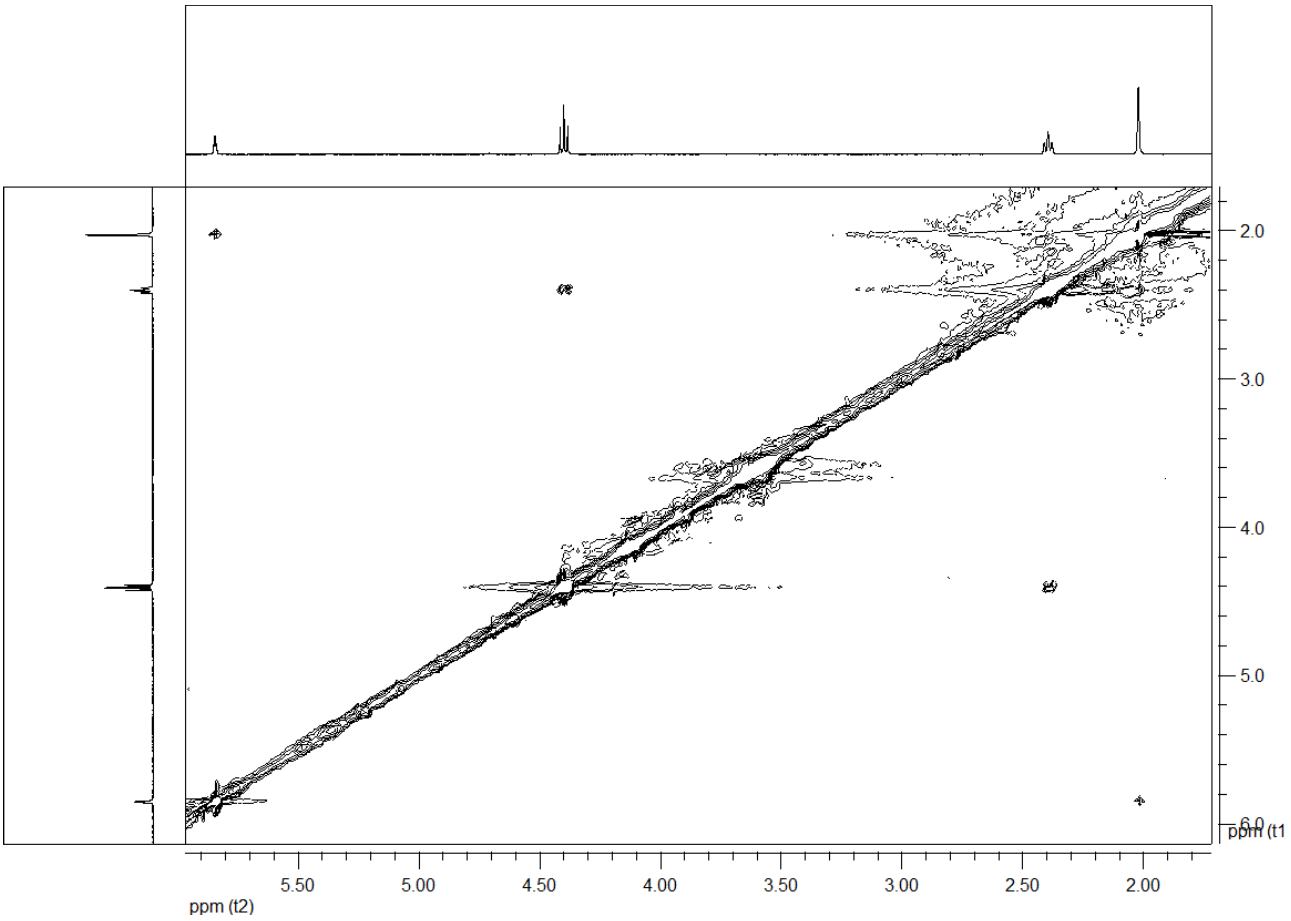

Figure S6. NOESY spectrum of talarodiolide (**1**) recorded in $CDCl_3$ at 400 MHz

Table S1: Mass spectra of identified metabolites. RI represents the Kovats non isothermal retention index on HP5MS 30 m capillary column. Panel "MS peaks annotations" reports mass, formula (in square brackets) and normalized abundance (in round brackets) of identified fragments in mass spectra; the molecular ion is always represented by M; Me indicates methyl (-CH_3). Abundance is normalized taking abundance of base peak equal 100; for instance array 224 [M]$^+$ (5) represents a molecular ion with integer mass of 224 Dalton present in a mass spectrum at an abundance 5% the abundance of base peak; analogously array 209 [M-Me]$^+$ (4) represents ion of mass 209 Dalton and abundance 4% which has been formed from the molecular ion by loss of a methyl fragment (15 Dalton).

Talarodiolide **(1)**	RI: 2064	MS peaks annotations:<br>224 [M]$^{\bullet+}$ (5), 209 [M-Me]$^+$ (4), 194 [M-2Me]$^+$ (35), 149 [M-2Me-CO_2-O]$^+$ (60), 70 [M-$C_8H_9O_3$]$^+$ (100)

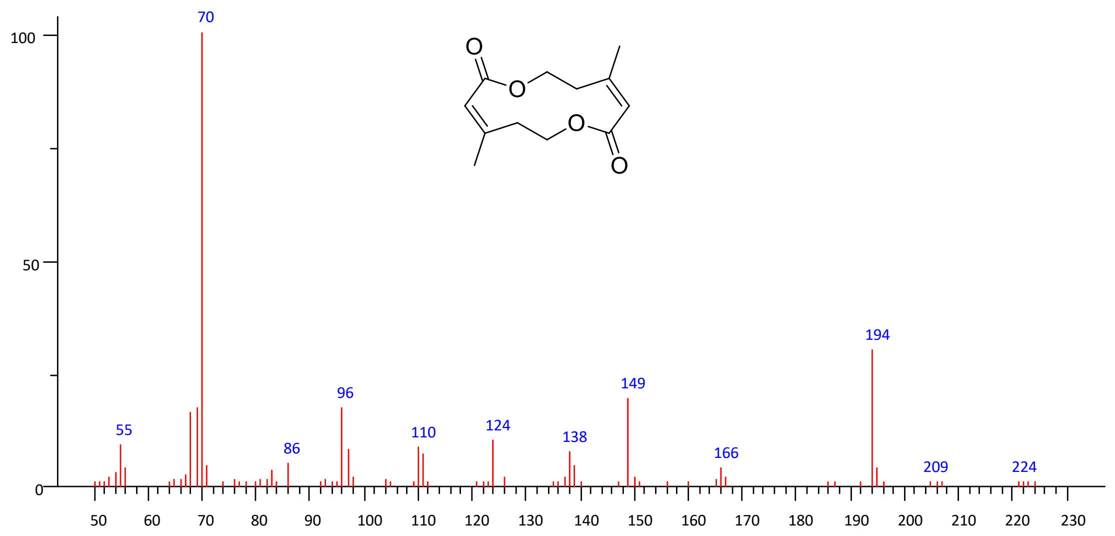

3-*O*-methylfunicone **(2)**	RI: 3006	MS peaks annotations:<br>388 [M]$^{\bullet+}$ (40), 373 [M-Me]$^+$ (15), 357 [M-2Me]$^+$, 223 [M-$C_9O_3H_9$]$^+$ (65), 192 [M-2Me- $C_9O_3H_9$]$^+$ (100)

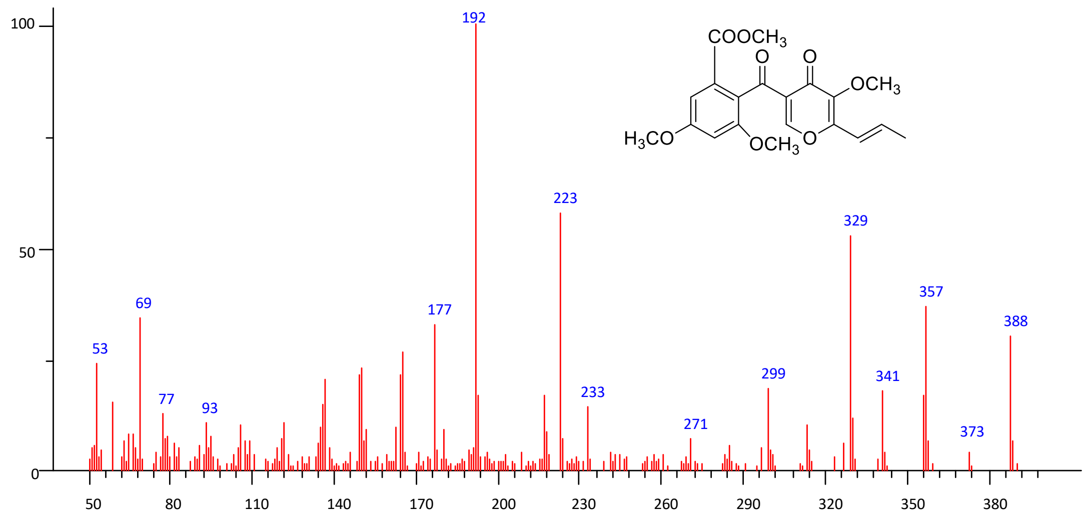

Cyclo-(Pro-Leu) **(3)**	RI: 2068	MS peaks annotations:<br>195 $[M-Me]^+$ (5), 154 $[M-C_4H_9]^+$ (100), 125 $[M-C_6H_{13}]^+$ (15), 111 $[M-C_7H_{15}]^+$ (3), 70 $[M-C_7NO_2H_{11}]^+$ (75)

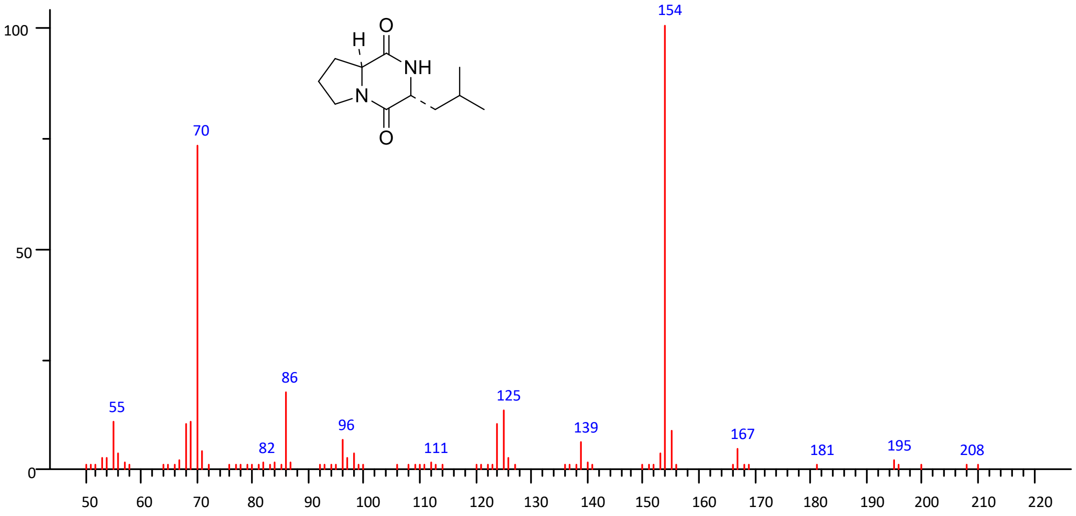

Cyclo-(Pro-Ile) **(4)**	RI: 2039	MS peaks annotations:<br>154 $[M-C_4H_9]^+$ (100), 125 $[M-C_6H_{13}]^+$ (120), 111 $[M-C_7H_{15}]^+$ (5), 70 [M-$C_7NO_2H_{11}]^+$ (65)

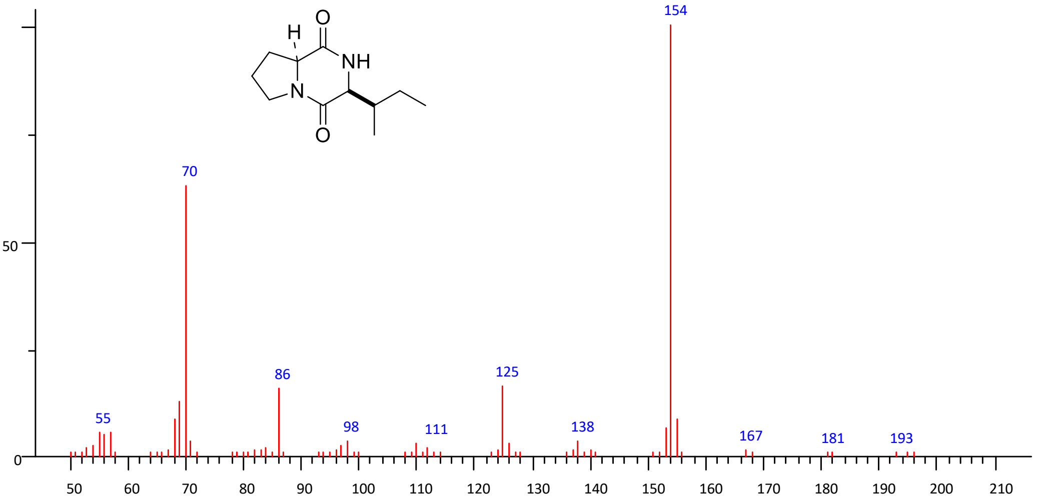

Cyclo (Pro-Phe) **(5)**	RI: 2443	MS peaks annotations:<br>244 [M]$^{\bullet+}$ (32), 215 [M-C_2H_4]$^+$ (3), 153 [M- C_7H_7] (28), 125 [M-C_3H_6-C_6H_5] (100)

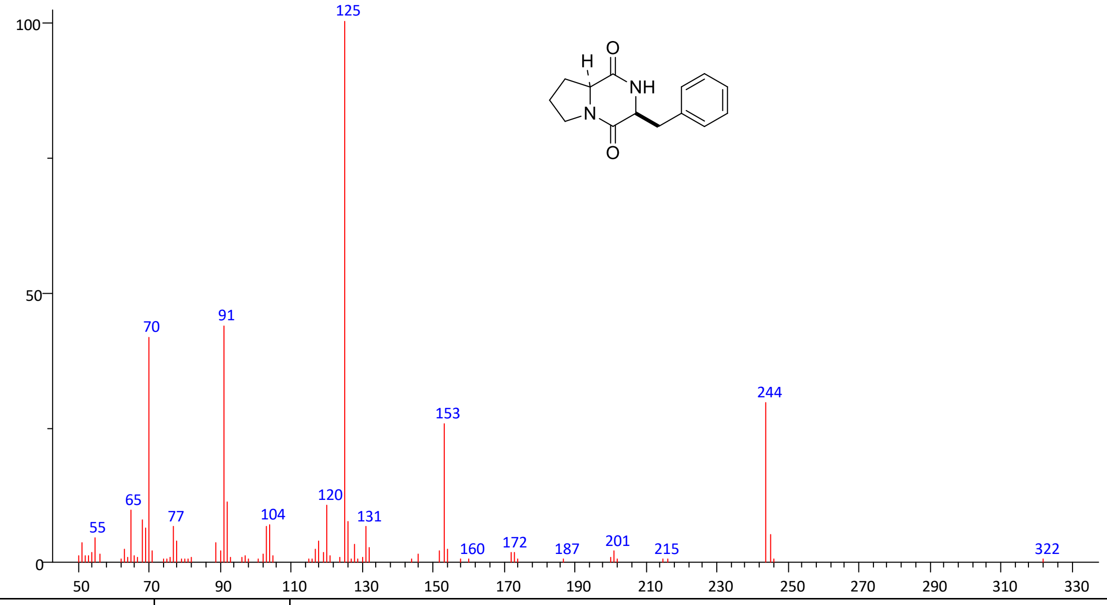

Vermistatin **(6)**	RI: 3105	MS peaks annotations:<br>328 [M]$^{\bullet+}$ (100), 313 [M-Me]$^+$ (10), 285 [M-Me-C_2H_4]$^+$ (48), 165 [M-C_2H_4-$C_8O_2H_8$]$^+$ (43)

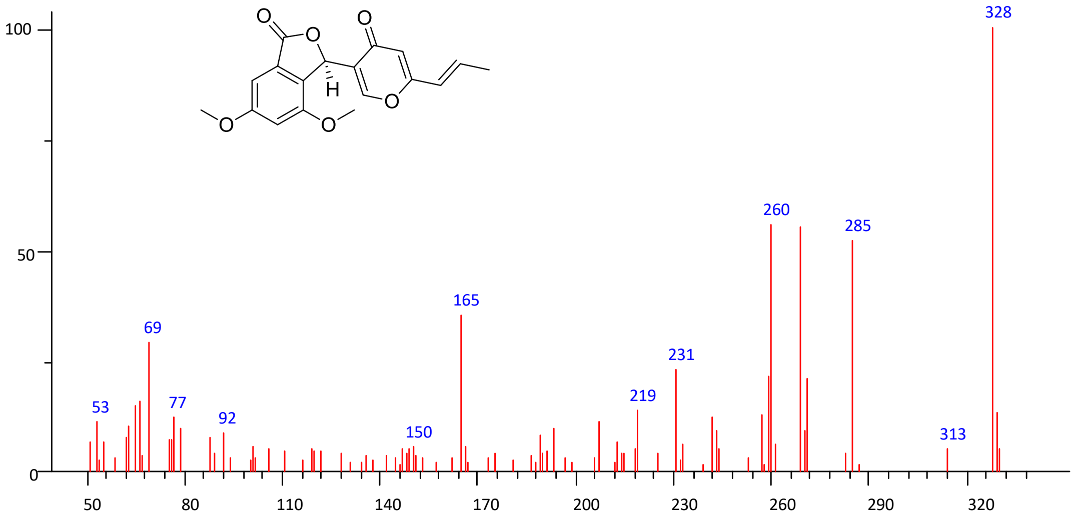

Penisimplicissin **(7)**	RI: 2835	MS peaks annotations:<br>302 $[M]^{\bullet+}$ (100), 287 $[M\text{-}Me]^{+}$, 273 $[M\text{-}2Me]^{+}$ (17), 175 $[M\text{-}Me\text{-}C_6H_7O_2]$ (14), 165 $[M\text{-}C_8O_2H_8]^{+}$ (47)

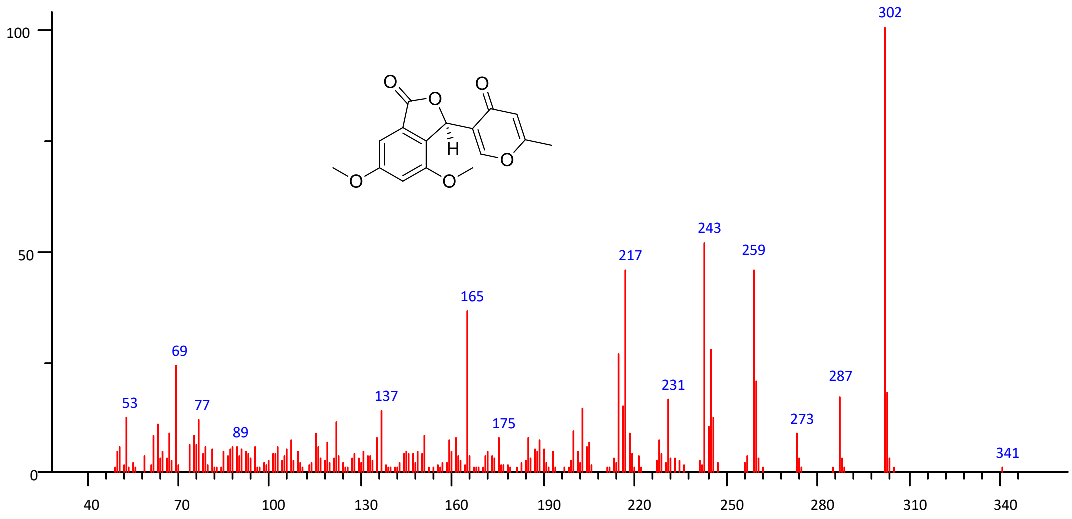

Penicillide **(8)**	RI: 3103	MS peaks annotations:<br>372 $[M\text{-}Me]^{+}$ (16), 269 $[M\text{-}2Me\text{-} C_5OH_{10}]$ (100), 253 $[M\text{-}Me\text{-}OCH_3\text{-}C_5OH_{10}]$ (20)

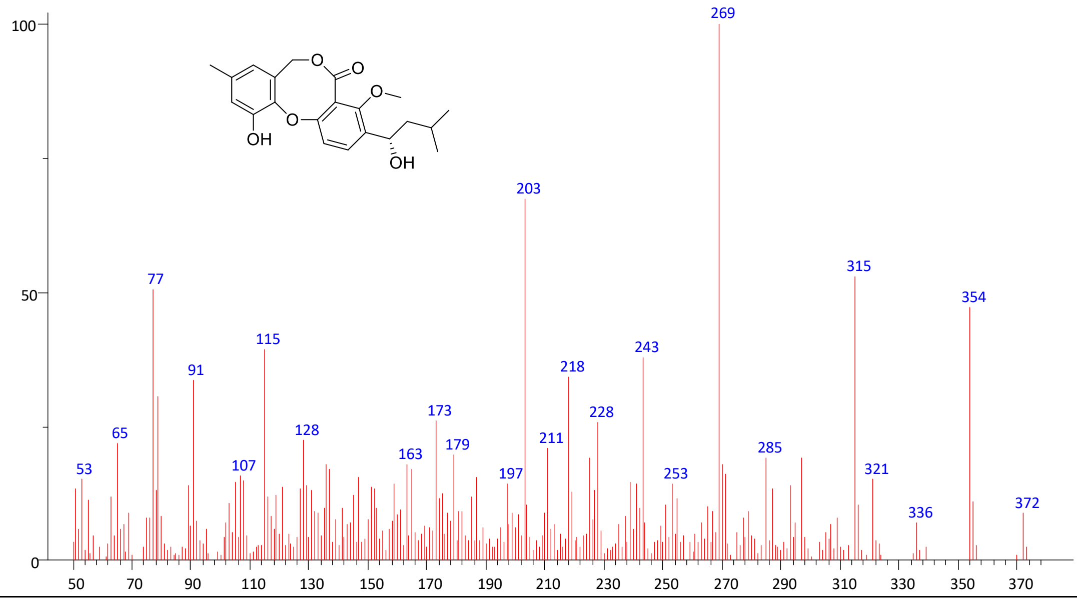

1-glycerol-linoleate **(9)**	RI: 2076	MS peaks annotations:<br>354 [M]•+ (4), 336 [M-OH]+, 262 [M-$C_3O_3H_7$]+ (63), 234 [M-$C_4O_4H_7$]+ (12)

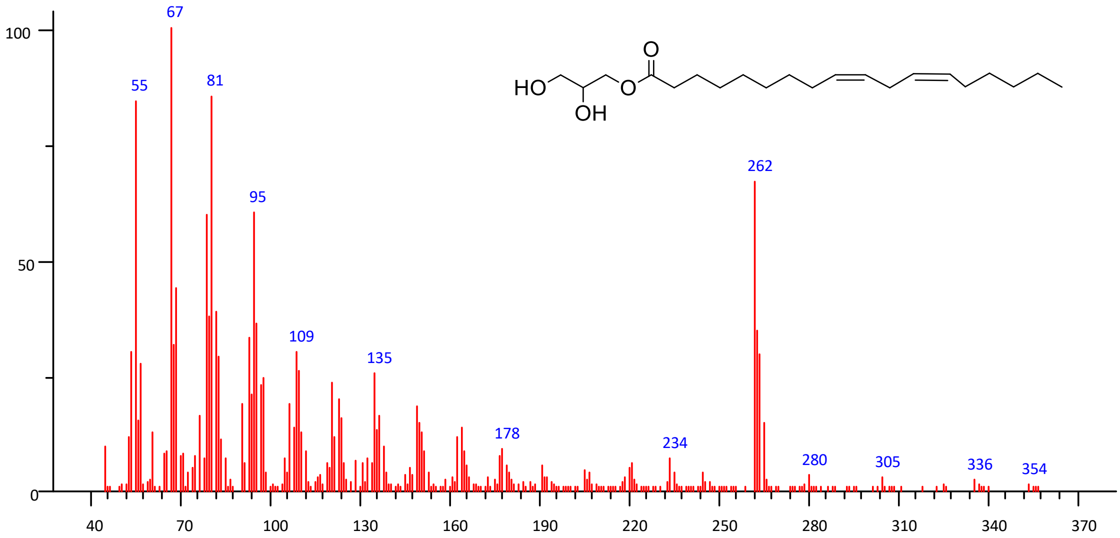

Supplement: Supplementary file 1 [file molecules-23-00950-s001.pdf]
